# Functional MYB transcription factor encoding gene *AN2* is associated with anthocyanin biosynthesis in *Lycium ruthenicum Murray*

**DOI:** 10.1186/s12870-019-1752-8

**Published:** 2019-04-29

**Authors:** Yuan Zong, Xuebing Zhu, Zenggen Liu, Xinyuan Xi, Guomin Li, Dong Cao, Le Wei, Jianming Li, Baolong Liu

**Affiliations:** 1 Qinghai Province Key Laboratory of Crop Molecular Breeding, Xining, 810008 China; 2 State Key Laboratory of Plateau Ecology and Agriculture, Qinghai University, Qinghai, Xining, 800010 China; 3 Key Laboratory of Adaptation and Evolution of Plateau Biota (AEPB), Northwest Institute of Plateau Biology, Chinese Academy of Sciences, Qinghai, Xining, 810008 China; 4 College of Biologic and Geographic Sciences, Qinghai Normal University, Qinghai, Xining, 810008 China

**Keywords:** *L. Ruthenicum*, *L. barbarum*, Fruit color, Anthocyanin biosynthesis, *AN2*

## Abstract

**Background:**

*Lycium ruthenicum Murray* is an important economic plant in China and contains higher levels of anthocyanins in its fruits than other *Lyciums*. However, the genetic mechanism of anthocyanin production in this plant is unknown.

**Results:**

Based on previous transcriptome analysis, *LrAN2* and *LbAN2*, encoding MYB transcription factors, were isolated from *L. ruthenicum* and *L. barbarum*, respectively. Both genes contained two introns, encoded 257 amino acids with two-Aa difference, and carried the unabridged HTH-MYB, MYB-like DNA-binding, and SANT domains. In the phylogenetic trees, *LrAN2* and *LbAN2* were found to be closely related to *NtAN2*, which regulates anthocyanin biosynthesis in tobacco. Overexpression of *LrAN2* and *LbAN2* induced anthocyanin biosynthesis in all tissues of tobacco. The anthocyanin content in the leaves of transgenic lines with *LbAN2* was lower than *LrAN2*. It indicated that the function of *LbAN2* was weaker than *LrAN2*. The *AN2* transcript could be detected only in the fruits of *L. ruthenicum* and increased during fruit development, accompanied by anthocyanin accumulation. In natural population, the alleles *LrAN2* and *LrAN2* were associated strictly with *L. ruthenicum* and *L. barbarum*, respectively*.* Moreover, an *AN2* genetic diversity study suggested that *Lyciums* with yellow, white, purple, and jujube red fruits were derived from *L. ruthenicum*.

**Conclusions:**

Two *AN2* alleles, from *L. ruthenicum* and *L. barbarum*, were functional MYB transcriptor regulating anthocyanin biosynthesis*.* The functional diversity and high expression level of *LrAN2* could be the reason for high anthocyanin content in the fruit of *L. ruthenicum*. *Lyciums* with yellow, white, purple, and jujube red fruits were derived from *L. ruthenicum* based on *AN2* sequence diversity. The results may be advantageous in identifying new varieties and breeding new cultivars.

**Electronic supplementary material:**

The online version of this article (10.1186/s12870-019-1752-8) contains supplementary material, which is available to authorized users.

## Background

*Lycium ruthenicum Murray* is a traditional Chinese herb listed in the Tibetan medical classic “Jing Zhu Ben Cao”. The fruit of *L. ruthenicum* has been used for the treatment of menopause, heart disease and abnormal menstruation for thousands of years in China [1]. The high anthocyanin content in the fruit of *L. ruthenicum* is the distinctness characteristically different to other *Lyciums,* which also has been thought to contributed to its special medicinal value [2–5]. Nowadays, the molecular mechanism of a high level anthocyanin synthesis remains unclear.

Generally, the structural genes for anthocyanin production included phenylalanine ammonia-lyase (*PAL*), Chalcone synthase (*CHS*), chalcone isomerase (*CHI*), flavonoid-3-hydroxylase (*F3H*), flavonoid-3′-hydroxylase (*F3’H*), and flavonoid-3′,5′-hydroxylase (*F3’5’H*) [6–11]. The structural genes are regulated by transcription factors such as V-myb avian myeloblastosis viral oncogene homolog (MYB) and basic Helix-Loop-Helix (bHLH) [12]. The inactivation of any of these structural genes and transcription factors blocks the metabolic pathway, causing plant tissues to display a pale phenotype [13]. However, allelic variations in the MYB and bHLH genes more commonly cause colour differentiation in plants. The promoter variation of functional *VvmybA1* is associated with the flesh pigmentation of intensely coloured grape varieties [14–16]. Similar MYB regulators have been identified in *Arabidopsis* (*MYB75*; *PAP1* and *AtMYB90*; *PAP2)* [17], petunia (*AN2*) [18] and sweet potato (*MYB1*) [19]. bHLH genes are also important for anthocyanin biosynthesis. The bHLH genes *R*, *B*, *Sn* and *Hopi* from maize could induce tissue-specific anthocyanin biosynthesis in maize, including expression in the aleurone, pericarp, anther, mesocotyl, root, leaf, and scutellum [20–24]. A 14-base-pair (bp) deletion within exon 6 that knocks out the bHLH domain of the protein RC causes white pericarps in rice [25]. Homologues of maize *R* and *B* genes were also found in *Antirrhinum* (*Delila*) [26], petunia (*Jaf13)* [27], and tomato (*ah*) [28].

Our previous research had identified the genes related to anthocyanin biosynthesis in the fruits of *L. ruthenicum* and *L. barbarum* based on transcriptome analysis. Compared with *L. barbarum,* 733,070 genes were upregulated while 25,779 genes appeared downregulated in the fruits of *L. ruthenicum.* All structural genes related to anthocyanin biosynthesis exhibited higher levels of expression in *L. ruthenicum* than *L. barbarum*, which implied the transcription factor was responsible for high anthocyanin content in the fruit of *L. ruthenicum*. The transcript level of bHLH genes in *L. ruthenicum* has no significant difference to *L. barbarum,* while the transcript level of the MYB transcription factor in *L. ruthenicum* was 35.66 times of *L. barbarum.* It could be induced that the MYB transcription factor played an important role in the black fruit formation of *L. ruthenicum.* Total four unigenes were homologous to the MYB transcription factor in the assembly sequence database, and these unigenes were thought to be derived from the same gene *AN2* after further sequence alignment.

In this manuscript, the MYB transcription factor gene *AN2s* were isolated from *L. ruthenium* and *L. barbarum* to evaluate its role in the black fruit development of *L. ruthenium*. It will lay a good molecular foundation for the selection of superior resources and breeding of new varieties of *L. ruthenicum*.

## Results

### Molecules characteristics

Previous experiments have shown that the expression of MYB transcription factor *AN2* occurs at a higher level in fruits of *L. ruthenicum* than *L. barbarum. LrAN2* and *LbAN2* were isolated from *L. ruthenicum* and *L. barbarum* based on RNA-sequence data. Both open reading frames (ORFs) of *LrAN2* and *LbAN2* are 774 bp in length and encode 257 amino acids. Although five nucleotide differences exist in the ORFs of *LrAN2* and *LbAN2*, only two amino acids differences have been discovered in translated sequences (Additional file 1: Figure S1). The nucleotide sequences of *LrAN2* and *LbAN2* contain 1383 bp and 1395 bp, respectively. Both contain two introns (Additional file 1: Figure S1), but 32 single nucleotide polymorphisms and one indel of 15 nucleotides in the second intron distinguish *LrAN2* from *LbAN2* (Additional file 1: Figure S1).

**Fig. 1:**
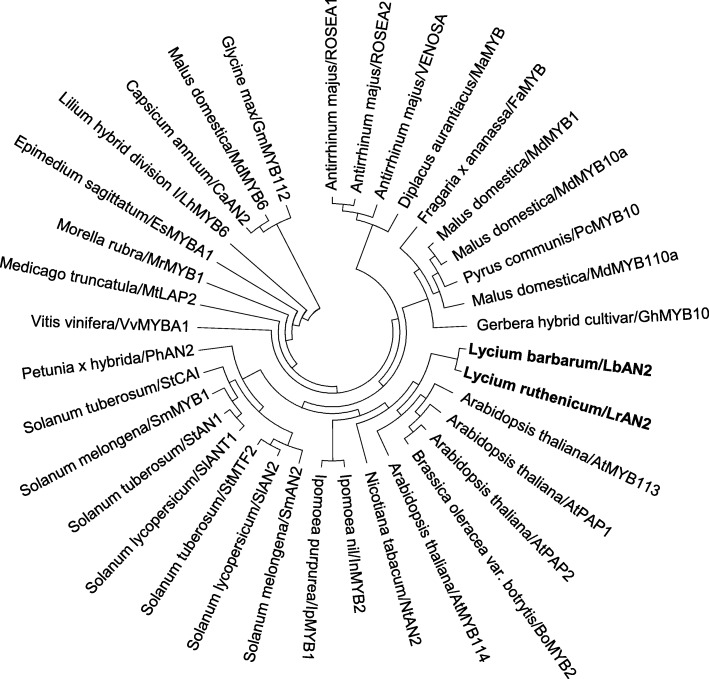
Phylogenetic relationship between *LrAN2*, *LbAN2* and anthocyanin-related MYB transcription factors in other species. The tree was constructed using MEGA6, neighbor-joining phylogeny testing and 1000 boot strap replicates. The accession number of these proteins (or translated products) follows the GenBank database: *Arabidopsis thaliana*/AtMYB113: NM_105308.2; *Arabidopsis thaliana*/AtPAP1: AB004318.1; *Arabidopsis thaliana*/AtPAP2: AB053950.1; *Brassica oleracea var. botrytis cultivar Stovepipe*/BOMYB2: GU219987.1; *Arabidopsis thaliana*/AtMYB114: NM_001334235.1; *Nicotiana tabacum*/NtAN2: FJ472647.1; I*pomoea nil*/InMYB2:AB234211.1; *Solanum melongena*/SmAN2: AGK37072; *Solanum lycopersicum*/SlAN2: ACT36603; *Solanum tuberosum*/StMTF2: ABY40371; *Solanum lycopersicum*/SlANT1: FJ705330.1; *Solanum tuberosum*/StAN1: JQ418343.1; *Solanum melongena*/SmMYB1: KT259043.1; *Solanum tuberosum*/StCAI:NM_001288113.1; *Petunia×hubrida*/PhAN2: AB982128.1; *Vitis vinifera*/VvMYBA1: AB097923.1; *Medicago truncatula*/MtLAP2: FJ199996.1; *Morella rubra*/MrMYB1: GQ340767.2; *Epimedium sagittatum*/EsMYBA1: KC335202.1; *Lilium hybrid division* I/LhMYB6: AB534587.1; *Capsicum annuum*/CaAN2: CAE75745; *Malus domestica*/MdMYB6: GU013682.1; *Glycine max*/GmMYB112: DQ822911.1; *Antirrhinum majus*/ROSEA1: KP311682.1; *Antirrhinum majus*/ROSEA2: DQ275530.1; *Antirrhinum majus*/VENOSA: DQ275531.1; *Diplacus aurantiacus*/MaMYB: KT355513.1; *Fragaria ananassa*/FaMYB: EU155162.1; *Malus domestica*/MdMYB1: GU013684.1; *Malus domestica*/MdMYB10a: AB744002.1; *Pyrus communis*/PcMYB10a: HM775223.1; *Malus domestica*/MdMYB110a: DQ074463.1; *Gerbera hybrid cultivar*/GhMYB10: EU130919.1

In order to determine the evolutionary relationship between Lycium *AN2* and MYB transcription factors in other plants that regulate anthocyanin biosynthesis, amino acid sequences were downloaded from the NCBI database to construct a phylogenetic tree. *LrAn2* and *LbAN2* were the closest to *NtAN2*, *AtMYB113*, *AtMYB114*, *AtPAP1* and *AtPAP2* (Fig. 1). *AN2* is clustered with other MYB transcription factors which were the main genes controlling anthocyanin synthesis in different tissues of other species. It includes many solanaceous plants, such as *Solanum melongena* (*SmAN2*), *Solanum lycopersicum* (*SlAN2*), *Solanum tuberosum* (*StMTF2*), *Petunia x hybrida* (*PhAN2*) and *Capsicum annuum* (*CaAN2*).

**Fig. 2:**
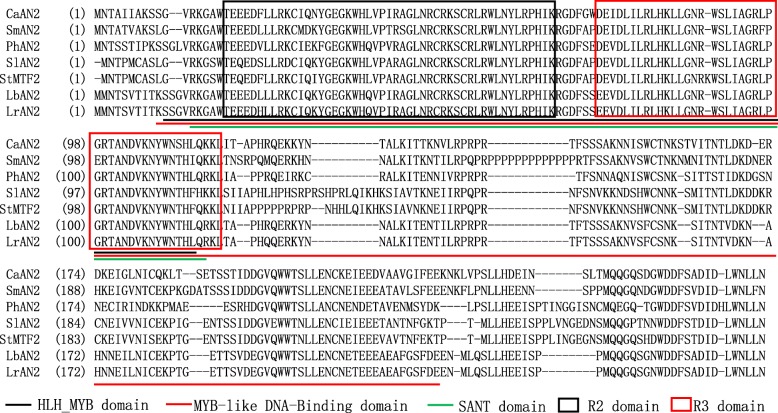
The alignment of the amino acid sequences of *LrAN2*, *LbAN2* and anthocyanin-related MYB transcription factors in other species. The three domains (HTH_MYB, MYB-like DNA-binding and SANT) that are conserved among known bHLH transcription factors regulating anthocyanin biosynthesis are underlined. R2 and R3 domains are outlined in black and red frames, respectively. The accession numbers of these proteins (or translated products) follows those in the GenBank database: *Capsicum annuum*/CaAN2: CAE75745; *Solanum melongena*/SmAN2: AGK37072; *Petunia×hubrida*/PhAN2: AB982128.1; *Solanum lycopersicum*/SlAN2: ACT36603; *Solanum tuberosum*/StMTF2: ABY40371

The amino acid sequences of *CaAN2*, *SmAN2*, *PhAN2*, *SlAN2*, and *StMTF2* were downloaded to investigate the structural domains of *LrAN2* and *LbAN2*. Both *LrAN2* and *LbAN2* contain the complete HTH_MYB, MYB-like DNA-binding, and SANT domains, which are important in regulating anthocyanin biosynthesis (Fig. 2). The two-Aa difference between *LrAN2* and *LbAN2*, L > H existed in the HLH domain and R > Q was just outside of the N terminus of MYB R3 domain.

### Transcription profile of *LrAN2* and *LbAN2*

**Fig. 3:**
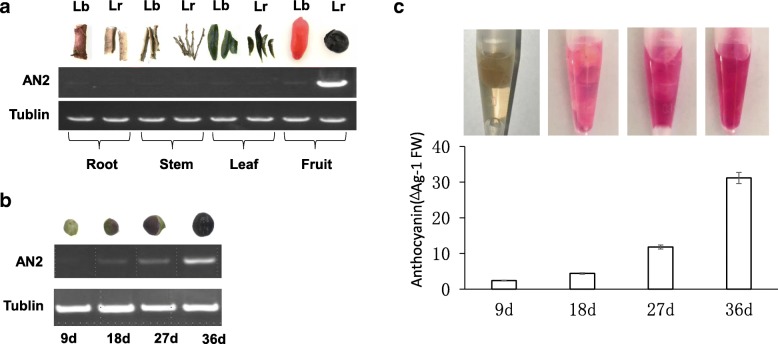
Transcription characteristics of *LrAN2* and *LbAN2*. **a** Relative transcript levels of *AN2* in root, stem, leaf and fruit of *L. barbarum* and *L. ruthenicum* as assessed using semi-quantitative RT-PCR. The amplification of the tubulin gene served as an internal control. **b** Relative transcript levels in the developing fruit of *L. ruthenicum*. The amplification of the tubulin gene served as an internal control. **c** Relative anthocyanin content in the developing fruit of *L. ruthenicum*

Semi quantitative PCR was used to compare the level of transcription for *LrAN2* and *LbAN2* in different tissues of *L. ruthenicum* and *L. barbarum*. The fruits of *L. ruthenicum* and *L. barbarum* take 36 days to develop. Fruit samples were collected at 9-day intervals for RT-PCR. The results showed that the *LrAN2* transcript could be detected only in the fruit of *L. ruthenicum* and was absent from root, stem, leaf and fruit of *L. barbarum* (Fig. 3a). Correspondingly, only the fruit of *L. ruthenicum* accumulated anthocyanin. Interestingly, the expression of *LrAN2* in *L. ruthenicum* increased with fruit development and was accompanied by anthocyanin accumulation (Fig. 3b, c).

### Overexpression of *AN2* induces anthocyanin biosynthesis in tobacco

**Fig. 4:**
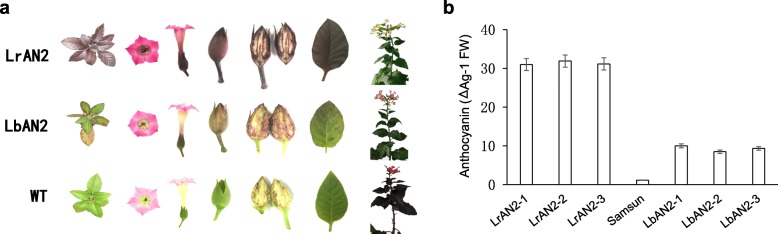
The phenotypes and relative anthocyanin contents of transgenic tobacco lines containing *LrNA2* and *LbAN2*. **a** phenotype of transgenic lines. **b** relative anthocyanin content of transgenic lines

The PJAM1502 constructs contained the double 35 s promoter which could drive the objective gene transcript in all tissues. Both transgenic lines, *LrAN2* and *LbAN2*, could induce anthocyanin biosynthesis in root, stem, leaf, flower and seed of tobacco (Fig. 4a). The transgenic lines of *LrAN2* showed the deeper purple leaf, while the transgenic lines of *LbAN2* displayed the plaques purple leaf (Fig. 4a). The relative anthocyanin content of the *LrAN2* transgenic lines was about 30 g^− 1^ fresh weight, while that of *LbAN2* was 10 g^− 1^ fresh weight (Fig. 4b). All transgenic lines contained higher anthocyanin content than the wild lines (Fig. 4b). These results show that both *LrAN2* and *LbAN2* can regulate anthocyanin biosynthesis by encoding MYB transcription factors.

### Allelic variation of *AN2* in natural populations of *Lycium*

**Fig. 5:**
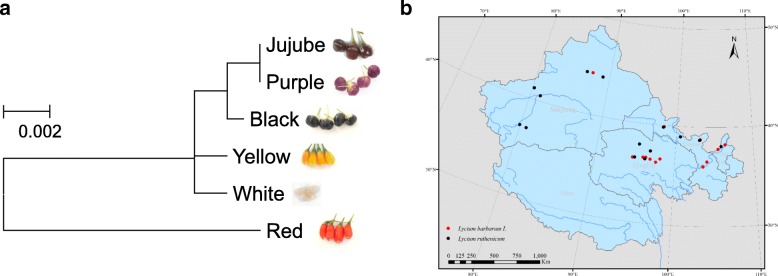
The phylogenetic tree of *AN2* alleles in *Lycium* (**a**) and the geographical distribution of *L. barbarum* and *L. ruthenicum* (**b**). The tree was constructed using MEGA6, neighbor-joining phylogeny testing and 1000 boot strap replicates

Apart from red fruit (*L. barbarum*) and black fruit (*L. ruthenicum*) varieties, some mutated lines of the *Lycium* family, which are restricted to particular regions, carry yellow, jujube, purple, and white fruit. To explore the genetic relationship between *L. ruthenicum, L. barbarum*, and these mutated lines, the genomic sequences of homologous *AN2* were isolated from the lines with yellow, jujube, purple, and white fruits. Only *LbAN2* from red fruit (*L. barbarum*) carried the same insertion of 15 nucleotides (Fig. 5a, Additional file 1: Figure S1). The mutation lines were determined to be at the same phylogenetic branch with Black fruit, which indicates that they are derived from *L. ruthenicum*.

A total of 54 *L. ruthenicum* and *L. barbarum* lines were collected from Qinghai, Xinjiang, Gansu and Ningxia, which are the main areas of distribution for *L. ruthenicum* and *L. barbarum* in China (Fig. 5b)*.* Based on the indel in the second intron of the nucleotide sequence, a set of special primers was designed to distinguish *LrAN2* from *LbAN2*. The special primers were effective in distinguishing *L. barbarum* from *L. ruthenicum* and other lines in China. All 24 *L. ruthenicum* lines were of the genotype *LrAN2* and the 30 *L. barbarum* lines were of the genotype *LbAN2* (Additional file 2: Table S1).

## Discussion

In this study, we isolated two alleles *LrAN2* and *LbAN2* from *L. ruthenicum* and *L. barbarum*, respectively, and investigated the function of *AN2* in regulating anthocyanin biosynthesis.

*LrAN2* is a functional MYB transcription factor gene regulating anthocyanin biosynthesis. Firstly, LrAN2 contained the complete HTH_MYB, MYB-like DNA-binding, which was important for exercising regulating function as a MYB transcription factor. In phylogenetic tree, LrAN2 was the same branch of MYB transcription factors (*NtAN2* and *AtPAP1*) [29, 30]. *NtAN2* gene encoded R2R3-MYB transcription factor and regulated anthocyanin synthesis in tobacco flowers [29]. While *AtPAP1* encoded *MYB75* transcription factor in *Arabidopsis thaliana*, which could induce purple anthocyanin production in most organs of *Arabidopsis thaliana* [30]. It implied LrAN2 was functional MYB transcriptor. Secondly, the transcript level of *LrAN2* was substantially higher in black fruits relative to red fruits and other tissues with low anthocyanin content. The fruit development of *L. ruthenicum* was accompanied by the increase of anthocyanin content and the expression of *LrAN2* gene. The transcript of *LrAN2* was relative to the anthocyanin biosynthesis obviously. Thirdly, the direct evidence was that overexpression of *LrAN2* promoted anthocyanin biosynthesis in tobacco, similarly to *NtAN2* and *AtPAP1*. Taken together, all results suggest that *LrAN2* is a functional analog of *NtAN2* encoding MYB transcription factors regulated to anthocyanin biosynthesis in plant cells [31–34].

Function diversity of *AN2* is possibly related to the production of black-colored fruit in *L. ruthenicum*. Thirty-two single nucleotide polymorphisms and one indel of 15 nucleotides in the second intron could differentiate *LrAN2* from *LbAN2*. In natural population, the alleles *LrAN2 and LrAN2* were associated strictly with *L. ruthenicum* and *L. barbarum*, respectively*.* Five nucleotide differences in the ORFs of *LrAN2* and *LbAN2* produce two amino acids differences*.* The two-Aa difference L > H existed in the HLH domain and R > Q existed just outside of the N terminus of MYB R3 domain. Theoretically, they were crucial determinants of protein structure and possibly function. The heterologous expression of *LrAN2* promoted anthocyanin biosynthesis in tobacco more effectively than *LbAN2*, which proved the function diversity exactly. Moreover, the transcript of *LrAN2* couldn’t been detect obviously in fruits of *L. barbarum*, which is consistent with our previous transcriptome experiment. Possible reason of high expression in *L. ruthenicum* could be the indels in their 2nd intron, because some indels could function in transcription regulation of anthocyanin biosynthesis. The promotor region could also contain some specific cis-recognition motif(s) to induce *LrAN2* transcript in the fruit of *L. ruthenicum*. Considering *LrAN2* was the only MYB transcription factor gene related to anthocyanin biosynthesis in black fruit of *L. ruthenicum* based on the transcriptome analysis, it could be inferred that both functional diversity and high expression level of *LrAN2* could be the reason for high anthocyanin content in the fruit of *L. ruthenicum*. More works were being enforced to prove the role of *LrAN2* in black fruit formation of *L. ruthenicum*.

## Conclusion

In this study, two allelic genes *LrAN2* and *LbAN2* were isolated from *L. ruthenicum* and *L. barbarum*. They carried the function regulating anthocyanin biosynthesis as the MYB transcription factors*.* The functional diversity and high expression level of *LrAN2* could be the reason for high anthocyanin content in the fruit of *L. ruthenicum*. *Lyciums* with yellow, white, purple, and jujube red fruits were derived from *L. ruthenicum* based on *AN2* sequence diversity. The results may be advantageous in identifying new varieties and breeding new cultivars.

## Methods

### Plant material

The *L. ruthenicum* variety LMH1 and *L. barbarum* variety Ningqi 7 are planted widely in China and the two cultivars were chosen for this research as representatives of *L. ruthenicum* and *L. barbarum*. Twenty-four wild varieties of *L. ruthenicum* and 30 wild varieties of *L. barbarum*, which were collected from different provinces in Northwest China, including Xinjiang, Qinghai, Gansu and Ningxia, were used to study the relationship between fruit color and presence of the *AN2* allele (Additional file 2: Table S1). Wolfberries in the colors purple, white, jujube red and yellow, from the genetically diverse area of the Qaidam basin, were used to study genetic variation of *AN2*. No permission was required in collecting the plants. Zenggen Liu is responsible for identifying and numbering these materials. All materials were preserved in the Northwest Plateau Institute of Biology, Chinese Academy of Sciences.

### DNA and cDNA preparation

One g of stem tip leaves was selected to extract genomic DNA [35]. Root, stem, leaf, and fruit samples were collected from corresponding plants for RNA extraction. The Trizol total RNA extraction method was used for extracting Total RNA [36]. A reverse transcription kit (Thermo Fisher First Strand cDNA Synthesis Kit, Beijing, China) was used according to the instructions to synthesize a cDNA from RNA.

### PCR and semi-quantitative PCR

Primers were synthesized by BGI Biological Technology Co., Ltd. The 50 μl reaction system included 10 μl 5×GC Buffer, 4 μl 10 mmol dNTP, 0.5 μl 20 pmol primer, and 0.5 μl (100 ng) cDNAs (Thermo Fisher Science, Beijing, China) were supplemented with ddH2O. The cycling conditions were as follows: 1 cycle at 98 °C for 2 min, 35 cycles at 98 °C for 10 s, 65 °C for 30 s and 72 °C for 2 min, followed by a cycle at 72 °C for 10 min. All PCR was conducted in the GeneAmp PCR System 9700 (Thermo-Fisher Scientific, Shanghai, China). The high-fidelity Phushion DNA polymerase (Thermo-Fisher Scientific, Shanghai, China)were used in all PCR reactions. The PCR products were extracted with the Tiangen TIANgel Midi Purification Kit (Tiangen) from 1.0% agarose gels and were cloned into the pGEM-T Easy Vector plasmid (Promega Corporation, Madison, Wisconsin, USA). The recombinant plasmid was transformed into *Escherichia coli* DH5α cells, and six positive clones were sequenced in a commercial company (Huada Gene, Shenzheng, China).

The semi-quantitative RT-PCR experiments were conducted using previously published methods [37]. Selecting of different tissue parts, including root, stem, leaf and fruit. The amplification of tubulin gene transcripts was used to normalize the cDNA contents of various reverse transcription mixtures before PCR and to monitor the kinetics of thermo-amplification during PCR. The reproducibility of the transcriptional patterns revealed by semi-quantitative PCR was tested by at least three independent assays. Additional file 3: Table S2 contained all primers used in this study.

### Overexpression of *LrAN2* and *LbAN2* in tobacco

The construct used for plant transformation was based on the binary vector *PJAM1502*, which contains a double 35 s promoter [38]. The construct *PJAM1502:LrAN2* and *PJAM1502:LbAN2* was based on the Gateway Cloning Kit (Invitrogen, USA). Binary vectors were electroporated into *Agrobacterium tumefaciens* strain GV3101. The leaf disc transformation method was used for tobacco transformation [39]. The selective medium of transgenic shoots contained 0.7% (*w*/*v*) agar, 3% (w/v) sucrose, 1.0 mg/L 1-Naphthaleneacetic acid (NAA), 1.0 mg/L 6-benzylaminopurine (BAP), 150 mg/L kanamycin, and 300 mg/L Timentin (ticarcillin disodium and clavulanate potassium). The transgenic shoots grow up in the greenhouse with long-day lighting (16 h light/8 h dark) after 1 month. For further experiments, the T3 family lines carrying objective gene without the separation were used.

### Relative anthocyanin content measurement

The fruits of 9, 18, 27 and 36 days after flowering were selected for estimating the anthocyanin content. The leaves of transgenic lines and wild lines were used for estimating the anthocyanin. Anthocyanin content was measured using the “Total Monomeric Anthocyanin Pigment Content of Fruit Juices, Beverages, Natural Colorants, and Wines” method (AOAC Official Method 2005.02) and calculated using data sets from three independent experiments. Statistical analyses of the obtained data were performed using the software package SPSS for Windows 11.5 with a 95% confidence interval [40, 41].

### Genotyping the natural population of *L. ruthenicum* and *L. barbarum* with *AN2sp*

To distinguish *L. ruthenicum* from *L. barbarum*, the polymorphic PCR marker, *AN2sp* was designed according to the nucleotide sequence difference between the second intron regions of *LrAN2* and *LbAN2*. The primers of *AN2sp* are listed in Additional file 3: Table S2. The amplicons produced by *AN2sp* were 117 bp in length for *LrAN2* and 132 bp in length for *LbAN2* (Additional file 4: Figure S2). The geographical distribution map is made by ArcGIS 10.0 software and base map is derived from ArcMap version 10.2.

### Bioinformatic analysis

Vector NTI 10 software (Thermo-Fisher Scientific, Waltham, MA) was used for The amino acid sequence alignment. Biological software (http://www.detaibio.com/sms2/translate.html) was used to translate the coding sequence into amino acids. The conservative functional domains were predicted using the website (http://www.ebi.ac.uk/interpro/). The amino acid sequence encoded by *LrAN2* and *LbNA2* was compared with other plant homologous sequences in GenBank by MEGA 6.0 software to determine whether *LrAN2* and *LbAN2* belong to the category of transcription factors controlling anthocyanin synthesis [42]. Finally, the oligonucleotide primers were designed by Primer 5 software (Premier Biosoft, Palo Alto, CA, USA).

## Additional files

Additional file 1: **Figure S1.** The alignment of nucleotide sequences of AN2 alleles from different Lycium. (DOCX 1579 kb)

Additional file 2: **Table S1.** The origin, AN2 genotype of the Lycium germplasm materials examined using AN2sp marker. (DOCX 18 kb)

Additional file 3: **Table S2.** Oligo nucleotide primers used in this work. (DOCX 13 kb)

Additional file 4: **Figure S2.** Development of the marker AN2sp for amplifying the two different alleles of AN2 (LrAN2 and LbAN2). (DOCX 175 kb)

## Abbreviations

BAP: 6-benzylaminopurine
bHLH: Basic Helix-Loop-Helix
CHI: Chalcone isomerase
CHS: Chalcone synthase
CoA: Co-enzyme A
F3’5’H: Flavonoid-3′,5′-hydroxylase
F3’H: Flavonoid − 3′- hydroxylase
F3H: Flavonoid-3-hydroxylase
NAA: 1-Naphthaleneacetic acid
ORFs: Open reading frames
RT-PCR: Quantitative real-time polymerase chain reaction
