# Supplementary material for: Functional MYB transcription factor encoding gene AN2 is associated with anthocyanin biosynthesis in Lycium ruthenicum Murray
Source: BMC Plant Biol. 2019 Apr 29;19:169. doi: 10.1186/s12870-019-1752-8 (PMC6489258; doi:10.1186/s12870-019-1752-8)
Supplement: Supplementary file 1 — Figure S1. The alignment of nucleotide sequences of AN2 alleles from different Lycium. (DOCX 1579 kb) [file 12870_2019_1752_MOESM1_ESM.docx]

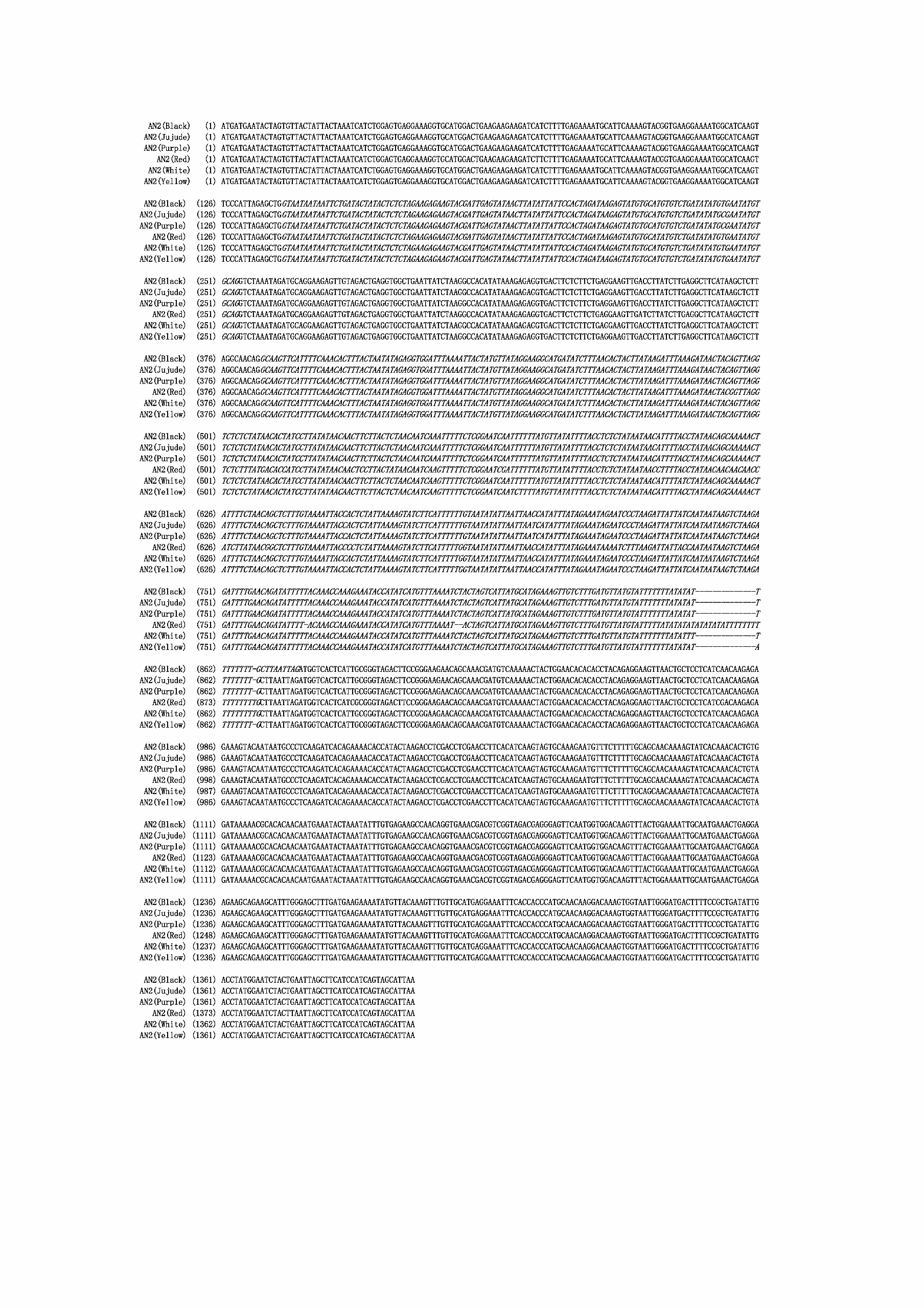


**Figure S1.** The alignment of nucleotide sequences of *AN2* alleles from different *Lycium*. The italic letters mean the intron region, and two introns exist in all *AN2* alleles. Only red fruit carried an insertion of 15 nucleotides in the second intron region of *AN2*.
