# Supplementary material for: Functional MYB transcription factor encoding gene AN2 is associated with anthocyanin biosynthesis in Lycium ruthenicum Murray
Source: BMC Plant Biol. 2019 Apr 29;19:169. doi: 10.1186/s12870-019-1752-8 (PMC6489258; doi:10.1186/s12870-019-1752-8)
Supplement: Supplementary file 2 — Table S1. The origin, AN2 genotype of the Lycium germplasm materials examined using AN2sp marker. (DOCX 18 kb) [file 12870_2019_1752_MOESM2_ESM.docx]

**Table S1.** The origin, AN2 genotype of the *Lycium* germplasm materials examined using *AN2sp* marker.

| Item | voucher number | Taxon | Province | City | Longitude  (East) | Latitude  (West) | Heigh  t(m) | Phenotype | Genotype |
| --- | --- | --- | --- | --- | --- | --- | --- | --- | --- |
| 1 | QTPMB-00031880 | L. barbarum | Qinghai | Geermu | 36°24.180 | 94°31.672 | 2790 | Red Fruit | LbAN2 |
| 2 | QTPMB-00031881 | L. barbarum | Qinghai | Geermu | 36°24.180 | 94°31.672 | 2790 | Red Fruit | LbAN2 |
| 3 | QTPMB-00031882 | L. barbarum | Qinghai | Dagele | 36°27.373 | 95°44.921 | 2780 | Red Fruit | LbAN2 |
| 4 | QTPMB-00031883 | L. barbarum | Qinghai | Dagele | 36°27.373 | 95°44.921 | 2780 | Red Fruit | LbAN2 |
| 5 | QTPMB-00031884 | L. barbarum | Qinghai | Nuomuhong | 36°24.611 | 96°14.377 | 2770 | Red Fruit | LbAN2 |
| 6 | QTPMB-00031885 | L. barbarum | Qinghai | Nuomuhong | 36°24.611 | 96°14.377 | 2770 | Red Fruit | LbAN2 |
| 7 | QTPMB-00031886 | L. barbarum | Qinghai | Balong | 36°08.150 | 97°31.413 | 2870 | Red Fruit | LbAN2 |
| 8 | QTPMB-00031887 | L. barbarum | Qinghai | Balong | 36°08.150 | 97°31.413 | 2870 | Red Fruit | LbAN2 |
| 9 | QTPMB-00031888 | L. barbarum | Qinghai | Dulan | 36°18.862 | 98°06.187 | 3170 | Red Fruit | LbAN2 |
| 10 | QTPMB-00031889 | L. barbarum | Qinghai | Dulan | 36°18.862 | 98°06.187 | 3170 | Red Fruit | LbAN2 |
| 11 | QTPMB-00031890 | L. barbarum | Qinghai | Delingha | 37°15.691 | 96°51.211 | 2810 | Red Fruit | LbAN2 |
| 12 | QTPMB-00031891 | L. barbarum | Qinghai | Delingha | 37°15.691 | 96°51.211 | 2810 | Red Fruit | LbAN2 |
| 13 | QTPMB-00031892 | L. barbarum | Gansu | Gaolan | 36°22.338 | 103°56.451 | 1730 | Red Fruit | LbAN2 |
| 14 | QTPMB-00031893 | L. barbarum | Gansu | Gaolan | 36°22.338 | 103°56.451 | 1730 | Red Fruit | LbAN2 |
| 15 | QTPMB-00031894 | L. barbarum | Gansu | Dongxiang | 35°43.887 | 103°25.577 | 2370 | Red Fruit | LbAN2 |
| 16 | QTPMB-00031895 | L. barbarum | Gansu | Dongxiang | 35°43.887 | 103°25.577 | 2370 | Red Fruit | LbAN2 |
| 17 | QTPMB-00031896 | L. barbarum | Gansu | Jiayuguan | 39°45.408 | 98°15.343 | 1730 | Red Fruit | LbAN2 |
| 18 | QTPMB-00031897 | L. barbarum | Gansu | Zhangye | 39°45.408 | 98°15.343 | 1730 | Red Fruit | LbAN2 |
| 19 | QTPMB-00031898 | L. barbarum | Gansu | Wuwei | 39°45.408 | 98°15.343 | 1730 | Red Fruit | LbAN2 |
| 20 | QTPMB-00031899 | L. barbarum | Gansu | Minqin | 38°37.359 | 103°06.699 | 1460 | Red Fruit | LbAN2 |
| 21 | QTPMB-00031900 | L. barbarum | Gansu | Minqin | 38°37.359 | 103°06.699 | 1460 | Red Fruit | LbAN2 |
| 22 | QTPMB-00031901 | L. barbarum | Ningxia | Zhongwei | 37°30.934 | 105°20.463 | 1230 | Red Fruit | LbAN2 |
| 23 | QTPMB-00031902 | L. barbarum | Ningxia | Zhongwei | 37°30.934 | 105°20.463 | 1230 | Red Fruit | LbAN2 |
| 24 | QTPMB-00031903 | L. barbarum | Ningxia | Wuzhong | 38°00.728 | 106°10.701 | 1190 | Red Fruit | LbAN2 |
| 25 | QTPMB-00031904 | L. barbarum | Ningxia | Lingwu | 38°00.728 | 106°10.701 | 1190 | Red Fruit | LbAN2 |
| 26 | QTPMB-00031905 | L. barbarum | Ningxia | Qingtongxia | 38°00.728 | 106°10.701 | 1190 | Red Fruit | LbAN2 |
| 27 | QTPMB-00031906 | L. barbarum | Xingjiang | Changji | 44°01.686 | 87°19.813 | 580 | Red Fruit | LbAN2 |
| 28 | QTPMB-00031907 | L. barbarum | Xingjiang | Changji | 44°01.686 | 87°19.813 | 580 | Red Fruit | LbAN2 |
| 29 | QTPMB-00031908 | L. barbarum | Xingjiang | Akesu | 40°36.346 | 80°50.325 | 1050 | Red Fruit | LbAN2 |
| 30 | QTPMB-00031909 | L. barbarum | Xingjiang | Akesu | 40°36.346 | 80°50.325 | 1050 | Red Fruit | LbAN2 |
| 31 | QTPMB-00031910 | L. ruthenicum | Qinghai | Geermu | 36.47° | 94.95° | 2782 | Black Fruit | LrAN2 |
| 32 | QTPMB-00031911 | L. ruthenicum | Qinghai | Geermu | 36.47° | 94.95° | 2782 | Black Fruit | LrAN2 |
| 33 | QTPMB-00031912 | L. ruthenicum | Qinghai | Geermu | 36.47° | 94.95° | 2782 | Black Fruit | LrAN2 |
| 34 | QTPMB-00031913 | L. ruthenicum | Qinghai | Nuomuhong | 36.41° | 96.24° | 2770 | Black Fruit | LrAN2 |
| 35 | QTPMB-00031914 | L. ruthenicum | Qinghai | Nuomuhong | 36.41° | 96.24° | 2770 | Black Fruit | LrAN2 |
| 36 | QTPMB-00031915 | L. ruthenicum | Qinghai | Nuomuhong | 36.41° | 96.24° | 2770 | Black Fruit | LrAN2 |
| 37 | QTPMB-00031916 | L. ruthenicum | Qinghai | Delingha | 37.26° | 96.85° | 2814 | Black Fruit | LrAN2 |
| 38 | QTPMB-00031917 | L. ruthenicum | Qinghai | Delingha | 37.26° | 96.85° | 2814 | Black Fruit | LrAN2 |
| 39 | QTPMB-00031918 | L. ruthenicum | Qinghai | Dacaidan | 37.84° | 95.36° | 3147 | Black Fruit | LrAN2 |
| 40 | QTPMB-00031919 | L. ruthenicum | Qinghai | Dacaidan | 37.84° | 95.36° | 3147 | Black Fruit | LrAN2 |
| 41 | QTPMB-00031920 | L. ruthenicum | Xingjiang | Alaer | 40.53° | 81.29° | 1080 | Black Fruit | LrAN2 |
| 42 | QTPMB-00031921 | L. ruthenicum | Xingjiang | Wensu | 41.13° | 80.21° | 1135 | Black Fruit | LrAN2 |
| 43 | QTPMB-00031922 | L. ruthenicum | Xingjiang | Wensu | 41.13° | 80.21° | 1135 | Black Fruit | LrAN2 |
| 44 | QTPMB-00031923 | L. ruthenicum | Xingjiang | Moyu | 37.11° | 79.91° | 1432 | Black Fruit | LrAN2 |
| 45 | QTPMB-00031924 | L. ruthenicum | Xingjiang | Cele | 37.01° | 80.80° | 1456 | Black Fruit | LrAN2 |
| 46 | QTPMB-00031925 | L. ruthenicum | Xingjiang | Qitai | 43.99° | 89.05° | 761 | Black Fruit | LrAN2 |
| 47 | QTPMB-00031926 | L. ruthenicum | Xingjiang | Hutubi | 44.18° | 86.70° | 584 | Black Fruit | LrAN2 |
| 48 | QTPMB-00031927 | L. ruthenicum | Xingjiang | Hutubi | 44.18° | 86.70° | 584 | Black Fruit | LrAN2 |
| 49 | QTPMB-00031928 | L. ruthenicum | Gansu | Jiayuguan | 39.80° | 98.23° | 1715 | Black Fruit | LrAN2 |
| 50 | QTPMB-00031929 | L. ruthenicum | Gansu | Jiayuguan | 39.80° | 98.23° | 1715 | Black Fruit | LrAN2 |
| 51 | QTPMB-00031930 | L. ruthenicum | Gansu | Zhangye | 38.94° | 100.56° | 1470 | Black Fruit | LrAN2 |
| 52 | QTPMB-00031931 | L. ruthenicum | Gansu | Minqin | 38.65° | 103.08° | 1363 | Black Fruit | LrAN2 |
| 53 | QTPMB-00031932 | L. ruthenicum | Ningxia | Qingtongxia | 38.03° | 105.86° | 1211 | Black Fruit | LrAN2 |
| 54 | QTPMB-00031933 | L. ruthenicum | Ningxia | Qingtongxia | 38.03° | 105.86° | 1211 | Black Fruit | LrAN2 |
