# Supplementary material for: Functional MYB transcription factor encoding gene AN2 is associated with anthocyanin biosynthesis in Lycium ruthenicum Murray
Source: BMC Plant Biol. 2019 Apr 29;19:169. doi: 10.1186/s12870-019-1752-8 (PMC6489258; doi:10.1186/s12870-019-1752-8)
Supplement: Supplementary file 3 — Table S2. Oligo nucleotide primers used in this work. (DOCX 13 kb) [file 12870_2019_1752_MOESM3_ESM.docx]

**Table S2.** Oligo nucleotide primers used in this work.

| **Primer** | **Sequence (5'-3')** | **Function** |
| --- | --- | --- |
| AN2cdsF | TGTTCTTAATGCTACTGATGG | Cloning *AN2* cDNAs |
| AN2cdsR | ATGATGAATACTAGTGTTACTAT |  |
| Tubulin-F | CCATACCAGCATCACCATTCTTC | Amplifying wheat tubulin gene transcripts (as internal control of RT-PCR) |
| Tubulin-R | GTCACACTTCCCACATTGCC |  |
| AN2-RT-F | ATGATGAATACTAGTGTTACTATTA | Amplifying *AN2* gene transcripts |
| AN2-RT-R | AGTCTACAACTCTTCCTG |  |
| AN2spf | ACTAGTCATTATGCATAGAAAGTTG | Amplifying the *AN2* difference in gDNA |
| AN2spr | CGTTTGCTGTTCTTCCCG |  |
| AN2attb1 | AAAAAGCAGGCTTCATGATGAATACTAGTGTTAC | Construction of pUbi-LrAN2, pUbi-LbAN2 |
| AN2attb2 | AGAAAGCTGGGTCCTAATTCAGTAGATTCCATA |  |
| Attb1 adapter | GGGGACAAGTTTGTACAAAAAAGCAGGCT | Universal *att*B adapter primers |
| Attb2 adapter | GGGGACCACTTTGTACAAGAAAGCTGGGT |  |
