# Supplementary material for: Functional MYB transcription factor encoding gene AN2 is associated with anthocyanin biosynthesis in Lycium ruthenicum Murray
Source: BMC Plant Biol. 2019 Apr 29;19:169. doi: 10.1186/s12870-019-1752-8 (PMC6489258; doi:10.1186/s12870-019-1752-8)
Supplement: Supplementary file 4 — Figure S2. Development of the marker AN2sp for amplifying the two different alleles of AN2 (LrAN2 and LbAN2). (DOCX 175 kb) [file 12870_2019_1752_MOESM4_ESM.docx]

**Figure S2.** Development of the marker *AN2sp* for amplifying the two different alleles of *AN2* (*LrAN2* and *LbAN2*). The fragments yielded by the marker were either 117 or 132 bp, which were indicative of *LrAN2* and *LbAN2,* respectively.
